# The Emerging Role of *ESR1* Mutations in Luminal Breast Cancer as a Prognostic and Predictive Biomarker of Response to Endocrine Therapy

**DOI:** 10.3390/cancers11121894

**Published:** 2019-11-28

**Authors:** Irene De Santo, Amelia McCartney, Ilenia Migliaccio, Angelo Di Leo, Luca Malorni

**Affiliations:** 1 Department of Clinical Medicine and Surgery, University of Naples Federico II, 80131 Naples, Italy; iredesanto@gmail.com; 2 “Sandro Pitigliani” Medical Oncology Department, Hospital of Prato, 59100 Prato, Italy; amelia.mccartney@uslcentro.toscana.it (A.M.); angelo.dileo@uslcentro.toscana.it (A.D.L.); 3 “Sandro Pitigliani” Translational Research Unit, Hospital of Prato, 59100 Prato, Italy; ilenia.migliaccio@uslcentro.toscana.it

**Keywords:** breast cancer, *ESR1* mutations, endocrine-resistance, liquid biopsy, prognostic and predictive biomarker, SERD

## Abstract

Mutations in the hotspot ligand-binding domain of the estrogen receptor (ER) gene *ESR1* have recently been recognized as mechanisms of endocrine resistance in endocrine receptor-positive metastatic breast cancer (MBC). Accumulating data suggest these mutations develop under the selective pressure of endocrine treatments, and are infrequent in untreated ER-positive breast cancers. In vitro studies show that these mutations confer ligand-independent activity, resistance to estrogen deprivation, and relative resistance to tamoxifen and fulvestrant. Post-hoc retrospective and prospective analyses of *ESR1* mutations in patients with MBC have consistently found that these mutations are markers of poor prognosis and predict resistance to aromatase inhibitors (AIs). These results warrant further investigation and prospective validation in dedicated studies. Moreover, studies are ongoing to clarify the activity of novel drugs in the context of metastatic endocrine resistant luminal breast cancer harboring *ESR1* mutations. In this review, we summarize the pre-clinical and clinical findings defining the characteristics of *ESR1* mutant breast cancer, and highlight the potential clinical developments in this field.

## 1. Introduction

Breast cancer (BC) is a complex disease that comprises different clinical and histopathological subtypes. Two-thirds of cases express estrogen receptor-α (ER) [1,2]. Several in vitro and in vivo studies have clarified the role of ER and its estrogen ligands in normal mammary gland development, as well as in breast cancer evolution [3,4,5]. *ESR1* is the gene that encodes ERα, a protein belonging to the nuclear receptor superfamily [6]. *ESR1* is composed of two activating function domains (AF-1, the N-terminal ligand independent portion, and AF-2, the C-terminal ligand-dependent portion), which regulate the transcriptional activity of the receptor, a ligand binding domain (LBD) located in the C-terminal part, a DNA-binding domain, and a hinge domain [7]. Upon ligand binding to the receptor, the engagement of co-regulatory proteins and binding to specific DNA motifs, such as estrogen responsive element (ERE) [8], is triggered to modulate the expression of genes fundamental to several processes, including tumorigenesis. ER-coregulatory complexes can also bind other transcription factors such as AP-1 and Nfk-B, in turn modulating their transcriptional activity [9,10]. This last transcriptional function of ER appears increased in ligand-independent conditions under growth factor stimulation [11]. Furthermore, ER interacts with different tyrosine kinase receptors and signaling proteins, activating their signaling pathways [12]. From a molecular point of view, ER-positive (ER+) BC presents two distinct phenotypes, which were originally defined by gene-expression profiling and for which clinical surrogates exist. Firstly, the more indolent luminal A-like subtype is characterized by low tumor grade, strong positive expression of ER and progesterone receptor (PgR), human epidermal growth factor receptor 2 (HER2)-negativity and a low proliferative index; and the luminal B-like subtype, which is typically more aggressive. Luminal B-like tumors express ER, but display variable and lesser degrees of ER/PgR expression, are HER2-negative, and are associated with high grade and/or high proliferative rate [13].

Resistance to endocrine therapy is a major challenge in the management of ER+/HER2-negative breast cancer. In the metastatic setting, the majority of these cancers initially respond to endocrine treatment, but almost ubquitiously eventually acquire resistance to antiestrogen drugs. Less frequently, de novo endocrine resistance is observed in approximately 15–20% of patients, with no or a short-lived initial response to endocrine therapy [14]. In the past 30 years, several research groups have proposed various mechanisms involved in acquired endocrine resistance [7]. Previous research efforts have investigated the relationship between ER expression/activity and sensitivity to endocrine therapy, implicating a multitude of mechanisms. Loss of ER expression leading to endocrine therapy insensitivity has been observed in 15–20% of metastatic BCs [10]. However, ER remains expressed in the majority of cases of BC with acquired endocrine resistance [7]. Several mechanisms may induce increased ER activity, including increased expression of ER [7] or its co-factors [7]. Importantly, the interaction between ER and growth factor receptor signaling (including crosstalk with HER2) or cellular kinase pathways (including MAPK, stress-related kinases, PI3K/AKT/mTOR, and CDK4/6 pathways) can modulate ER activity via phosphorylation of ER itself and/or its co-regulators, resulting in fundamental modification of ER nuclear activity, which ultimately leads to endocrine resistance [7,11]. Hyperactivation of such signaling pathways can result from genetic alterations in a number of different genes, including NF-1. Post-translational modifications of ER, including methylation, acetylation, and SUMOylation, have been linked to endocrine resistance; additionally, delocalization of the ER to the cellular membrane, enabling ER crosstalk with other proteins, including growth factor receptors and their interacting proteins, and G protein-coupled receptor 30 (GPR30) have been involved in the development of the endocrine-resistant phenotype. Other critical factors contributing to endocrine resistance involve the tumor microenvironment and immune landscape. A deep discussion of this complex milieu, which has been explored recently by others [7,10], is beyond the scope of this concise review, which focuses specifically on the role of *ESR1* mutations. 

Recently Razavi et al. combined genomic sequencing results of 1918 BC tumors (of which 1501 were ER+) with data pertaining to clinical and treatment outcomes [15]. Their findings suggested a potential new taxonomy for resistance to endocrine therapy observed following treatment, which sub-classifies endocrine-resistant disease into four groups: (1) Those harboring *ESR1* mutations, which represent about 18% of the tumors relapsing after endocrine therapy; (2) functional alterations in the MAPK pathway and (3) mutations in the machinery of transcriptional regulation (MYC/TF) (constituting 13% and 9% of resistant cases, respectively); and (4) pan-wild-type tumors with a still unknown mechanism of resistance to hormonal therapy (representing the remaining 60% of cases). These alterations may pre-exist in treatment-naïve disease, or be acquired under selective pressure of endocrine therapy, resulting in the expansion of pre-existing resistant clones. In this review, we focus on the pre-clinical and clinical studies investigating *ESR1* mutations in ER+/HER2-negative metastatic breast cancer (MBC), highlighting their potential prognostic and predictive roles, and how awareness of mutational *ESR1* status might impact upon clinical decision-making and the potential for biomarker discovery. A biomarker is defined as prognostic if it provides information about cancer outcome regardless of therapy, whereas a predictive biomarker is prospectively reflective of the effect of a therapeutic intervention [16].

## 2. *ESR1* Gene Alterations

Several studies have shown that acquired genetic alterations, including gene amplification, in key target genes may lead to adaptive resistance to targeted therapies [17,18,19,20]. Li et al. demonstrated that in an ER+ patient-derived xenograft (PDX) obtained from a patient with endocrine-resistant MBC, *ESR1* gene amplification led to ligand-independent tumor proliferation [21]. However, studies employing next generation sequencing (NGS) showed that *ESR1* amplification in either primary or metastatic tumor is quite rare, occurring at a rate below 2% [7,22], thus reflecting a minor role in endocrine resistance. The clinical relevance of *ESR1* gene amplifications in determining endocrine resistance is still unclear.

Genomic rearrangements of *ESR1* leading to the dysregulation of gene transcription and the production of fusion genes have also been related to endocrine resistance. Veeraraghavan et al. identified a frequent genomic rearrangement involving the *ESR1* and CCDC170 (YAP1) genes [23]. The fusion gene *ESR1*–CCDC170 demonstrates gain-of-function via the expression of N-terminally truncated CCDC170 under the constitutionally active promoter of the *ESR1* gene. The product of YAP1/*ESR1* translocation induces a reduced sensitivity to fulvestrant, due to the lack of LBD *ESR1* in this chimeric protein [23]. More recently, Hartmeier et al. have shown that N-terminal *ESR1* fusions involving exons 6–7 are rare recurrent events in metastatic BC with potential implications for clinical resistance to endocrine therapy [24]. New insights into the genomic complexity of breast cancer have been derived from large-scale genomic studies. Results from the pivotal Cancer Genome Atlas (TCGA) project demonstrates that primary luminal breast cancers exhibit a lower mutation rate than other intrinsic subtypes, including basal-like and HER2-enriched breast cancers. Furthermore, mutations with a frequency of more than 5% were found in only eight genes, namely PIK3CA, TP53, MAP3K1, MAP2K4, GATA3, MLL3, CDH1, and PTEN. Mutations in the *ESR1* gene were found to be relatively rare in primary BCs (<5%) [17]. Data from Karnink [25] and Roodi [26] are in line with TCGA results, suggesting that *ESR1* mutations are infrequent in primary BC. Contrastingly, research focusing on the genomic characterization of metastatic BC has shown that *ESR1* mutations are more frequent in advanced endocrine receptor positive disease, occurring at a frequency of 12% (9/76; 95% confidence interval (CI), 6–21%) in metastatic tumors. In a subgroup of patients who received an average of seven lines of treatment, the frequency was 20% (5/25; 95% CI, 7–41%) [22,27]. *ESR1* mutations tend to cluster in a hotspot region coding the ligand binding domain (LBD) of ER, changing amino acid 536, 537, or 538 in helix 12 (p.Leu536Arg, p.Tyr537Ser, p.Tyr537Asn, p.Tyr537Cys, and p.Asp538Gly) [22,28]. Toy at al. demonstrated that LBD *ESR1* mutations found in this hotspot region exhibit a constitutively ligand-independent ER activity, which activates ER transcription function, promoting hormone-independent tumor cell growth [29]. This and further studies have shown that this activation occurs in the heterozygous state, suggesting that the functional status of wild-type alleles are overridden by that of co-existing mutations [30]. The most common *ESR1* mutations are Y537S, D538G, and E380Q [31]. E380Q mutations gather outside *ESR1* LBD hotspot cluster sites, and exhibit a yet-to-be-understood mechanism of cancer proliferation different to that of the constitutive activation identified in Y537S and D538G mutants [22]. A pioneering study by Fuqua et al. reported another *ESR1* somatic mutation, K303R (Lys to Arg), which lodges in the ER hinge domain [32]. This mutation confers a higher sensitivity to estrogen, and a lower response to endocrine treatment in ER+ BC cells. K303R mutation was the first to be described in BC [32,33,34], but its clinical significance is still under evaluation.

## 3. Pre-Clinical Data: *ESR1* Mutations and Drug Resistance

Pre-clinical data show that BC cells harboring LBD *ESR1* mutations display partial resistance to tamoxifen and fulvestrant in vitro, as higher doses of these agents are needed to elicit their anti-proliferative effect in cells carrying such mutations [22,28]. Data also suggest that LBD *ESR1* mutations confer complete resistance to aromatase inhibitors [22]. This may be ascribed to the different mechanism of action of these drugs: Tamoxifen is a selective ER modulator (SERM) and fulvestrant is a selective ER down-regulator (SERD), both binding ER to modulate its activity. The affinity of tamoxifen and fulvestrant to *ESR1* LBD mutants has not yet been fully elucidated, but emerging evidence suggests that the conformational change induced by mutations reduces affinity, which may explain the partial resistance mechanism [21,35]. Conversely, aromatase inhibitors do not bind directly ER, but act by reducing the levels of the estrogen ligand. Therefore, they cannot inhibit the ligand-independent activity of the mutant ER. More detailed in vitro studies have shown that different *ESR1* mutations may have a differential impact on the response to fulvestrant treatment [29]. Utilizing xenograft models, it was observed that fulvestrant fully inhibited BC cells with both wild-type *ESR1*, and with the E380Q mutation, whilst BC cells with Y537S mutations were less inhibited by fulvestrant therapy. Indeed, in this model, it was observed that the Y537S mutant model needed higher levels of fulvestrant to completely inhibit its activity [36]. 

Wardell et al. [37] tested the activity of the cyclin-dependent kinase 4/6 (CDK 4/6) inhibitor palbociclib administered as both monotherapy or in combination with the SERM bazedoxifene, in PDX models derived from patients with ER+ endocrine-resistant BC. Palbociclib monotherapy showed activity in PDX with *ESR1* wild-type, and in PDX with *ESR1* amplification, but was ineffective in PDX with *ESR1* D538G mutation. However, this observation may be explained by the concurrent loss of Rb expression in this model, a well-described mechanism of resistance to CDK4/6 inhibitors. Conversely, in the *ESR1* Y537S mutant PDX model, palbociclib alone or in combination with bazedoxifene similarly inhibited tumor growth, but the combination proved more effective in decreasing Ki67 expression than either agent given as monotherapy [37].

## 4. *ESR1* Mutations in a Clinical Context: Difference Between Metastatic and Early Breast Cancer

Recently, Schiavon et al. analyzed *ESR1* mutations on circulating tumor DNA (ctDNA) from 171 patients affected by metastatic BC, and from 28 patients with early stage disease [38]. These data showed that *ESR1* mutations are often selected during treatment with aromatase inhibitors in the metastatic setting, whilst are rarely acquired during adjuvant therapy with aromatase inhibitors (AI) [38]. However, Kuang et al. observed higher rates of *ESR1* mutations in patients treated with AI regardless of the treatment setting (adjuvant, metastatic or both) [39]. In line with the concept that *ESR1* mutations are primarily acquired during aromatase inhibitors treatment, Allouchery et al. have recently published findings from 42 patients with early BC treated with AI for at least 2 years, showing that *ESR1* mutations were not found in any of the studied patients [40]. These data confirm prior observations that detecting *ESR1* mutations in early BC is a rare event, ranging between 2 and 5% depending on the dataset [22]. In a recent study, *ESR1* mutations were detected in only 2.7% of 73 cases of early BCs [41]. In the same study, the authors explored the correlation between the frequency of *ESR1* mutations detected in ctDNA in patients with metastatic disease (*N* = 68), and the number of treatment lines received in the metastatic setting. Contrastingly, the occurrence rate of *ESR1* mutations ranged between 25 and 43%, depending on the number of treatment lines received. Jeselsohn et al. recently reviewed studies reporting *ESR1* LBD mutations, describing a frequency of mutations of 21% in patients who received at least one line of endocrine therapy [22]. A series of studies focused on *ESR1* LBD-activating mutations demonstrated that these mutations are more frequent in samples from heavily pre-treated BC patients and, in particular, in those exposed to prior AI treatment [21,22,29]. Overall, these data suggest that *ESR1* mutations occur more frequently in the advanced setting than in early BC, that the frequency is higher in more heavily pre-treated endocrine-resistant patients, and that treatment with aromatase inhibitors may exert a selective pressure favoring the expansion of *ESR1*-mutated clones.

### 4.1. ESR1 Mutations in Advanced Luminal BC: Prognostic and Predictive Biomarker?

A recently published meta-analysis which evaluated 1530 patients with ER+/HER2-negative metastatic BC suggested that *ESR1* mutations detected in ctDNA may serve as a potential prognostic biomarker in the advanced setting, with *ESR1* mutations associated with worse progression-free survival (PFS) and overall survival (OS) [42]. Regarding specific *ESR1* mutations, the authors observed that the D538G mutation was associated with a worse prognosis and shorter PFS, whereas the Y537S mutation was not associated with poor PFS, regardless of treatment received. Furthermore, all hotspot *ESR1* mutations were predictive of resistance to aromatase inhibitors, but not to other endocrine therapies. 

BOLERO-2 is a phase III randomized trial which compared everolimus, an inhibitor of mammalian target of rapamycin (mTOR), plus exemestane, a steroidal aromatase inhibitor, versus exemestane plus placebo in patients with metastatic ER+ disease refractory to previous therapy with a non-steroidal aromatase inhibitor [43]. In a sub-analysis comparing the prevalence of cfDNA *ESR1* mutations and clinical outcome on BOLERO-2, D538G and Y537S mutations were found in 28.8% and 13.3% of the samples, respectively, with 6% of cases harboring both mutations [44]. Compared to wild-type, the presence of any of these mutations was associated with more aggressive disease and a decrease in OS, regardless of previous treatments (*ESR1* wild-type, OS = 31.1 months; D538G mutation, OS = 25.99 months; Y537S mutation, OS = 19.98 months; both mutations, OS = 15.5 months), confirming the prognostic role of mutational status in the metastatic setting. In the sequencing of primary tumors derived from 183 women enrolled in BOLERO-2, only 3% harbored an *ESR1* mutation; therefore, no additional analysis was possible to clarify the prognostic role of these mutations in primary tumors [45]. The incidence of *ESR1* mutations occurring in selected completed large clinical trials is presented in Table 1.

The prognostic role of *ESR1* mutations has been also demonstrated by Schiavon and colleagues, which showed that *ESR1* mutations correlated with a shorter PFS in patients exposed to a subsequent aromatase inhibitor treatment [38]. Interestingly, in this analysis, E380Q mutations, which occur outside *ESR1* LBD, were observed to confer less sensitivity to tamoxifen and fulvestrant treatment, similar to the Y537S mutant. Using droplet digital PCR (ddPCR), Clatot et al. observed *ESR1* D538, Y537S/N/C mutations in 30.6% of plasma samples derived from patients with metastatic BC which had progressed after first-line aromatase inhibitor therapy [46]. The presence of *ESR1* mutations was shown to be relative to the length of time exposed to aromatase inhibitors, wherein exposure was more prolonged in patients with *ESR1* mutations than in patients with wild-type *ESR1*. *ESR1* mutations were found before clinical progression to AI therapy in 75% of cases, suggesting that tracking *ESR1* mutations in ctDNA could be useful in following disease changes under the selective pressure of aromatase inhibitor therapy. Additional data have shown that increases in ctDNA *ESR1* mutations during targeted therapy may predict a shorter duration of post-endocrine treatment efficacy [45].

Two other groups performed a post-hoc analysis of *ESR1* mutations occurring in clinical trials, to further elucidate their predictive or prognostic value in ctDNA. The first study is the SoFEA trial, a phase III randomized controlled trial which compared fulvestrant with or without concomitant anastrozole to exemestane, in postmenopausal women with ER+/HER2-negative MBC, whose disease previously progressed on non-steroidal aromatase inhibitors [47]. In a secondary analysis of the SoFEA trial, *ESR1* mutations were detected in 39.1% of baseline plasma sample from 161 patients [48]. Patients with *ESR1* mutations receiving exemestane had a worse median PFS compared to those receiving fulvestrant-containing regimens (2.6 months versus 5.7 months; HR, 0.52, 95% CI 0.30–0.92, *p* = 0.02). Contrastingly, patients with wild-type *ESR1* had a similar median PFS when given exemestane or fulvestrant (interaction test between treatments and *ESR1* status *p* = 0.07). In another prospective plasma DNA AI study of 39/83 patients who progressed on first-line AI therapy for advanced disease, *ESR1* mutations were detected in plasma approximately 6.7 months before clinical progression on treatment was discerned [49]. A post-hoc analysis focused on detecting *ESR1* mutations in plasma samples derived from patients recruited in the FERGI study, a randomized, double-blind, placebo-controlled, phase II trial in which was tested the PI3K inhibitor pictilisib plus fulvestrant versus fulvestrant alone, in aromatase inhibitor-resistant ER+/HER2-negative MBC [50]. *ESR1* mutations were detected in 37% (57/153) of baseline samples of enrolled patients, particularly in those with luminal A BC and PIK3CA-mutated tumors. However, this analysis did not demonstrate an association between the presence of *ESR1* mutations and clinical outcome [51]. Collectively, these data suggest that *ESR1* mutations are predictive for resistance to AI treatment and have a negative prognostic value regardless of therapy.

### 4.2. ESR1 Mutations in Patients Treated with CDK4/6 Inhibitors

In a prospective analysis of 155 plasma samples derived from patients with advanced BC, with plasma samples collected at any time during metastatic disease, *ESR1* mutations detected by ddPCR were found to be less frequent in BC treated with CDK4/6 inhibitors in combination with fulvestrant (113 of 155 patients had ER+/ HER2-negative disease; 34 of these harbored an *ESR1* mutation, two patients treated with combination palbociclib and fulvestrant = 5.9%), than in patients treated with fulvestrant alone (32 treated with fulvestrant alone = 94.1%; *p* = 0.01) [39]. Of note, in this prospective analysis, baseline *ESR1* mutations were associated with resistance to prior aromatase inhibitor therapy [41]. The PALOMA-3 trial (NCT01942135) originally compared palbociclib plus fulvestrant versus placebo plus fulvestrant in women with metastatic disease which had progressed on previous endocrine therapy [52]. A recently published sub-analysis of PALOMA-3 examined ctDNA derived from 195 patients with paired baseline and end-of-treatment plasma samples [53]. Analysis showed a positive selection of *ESR1* Y537S mutation at the end of treatment in both arms, suggesting a role of this mutation in conferring resistance to fulvestrant therapy, but not to CDK4/6 inhibition. This in turn suggests that resistance to fulvestrant itself may be the mitigating factor of resistance to the combination of palbociclib and fulvestrant, further confirming *ESR1* Y537S mutation as a negative predictive biomarker of fulvestrant response. These data also suggest that palbociclib does not prevent selection of *ESR1* mutations, which is in line with previous data in metastatic BC patients treated with palbociclib and letrozole [54].

It has been shown that PFS in patients receiving fulvestrant plus palbociclib in PALOMA-3 was longer than those receiving fulvestrant alone, regardless of *ESR1* mutation status [53]. Additionally, patients with *ESR1* mutations treated with the combination had a better outcome than patients with *ESR1* mutations receiving fulvestant monotherapy, suggesting that mutational status had a prognostic but not predictive value. In further support of their prognostic role, baseline tumor *ESR1* mutation rates were found to be lower among long-term responders in both PALOMA-3 trial arms [55]. A recent analysis of plasma samples collected at baseline, cycle 1 day 15, and at the end of treatment (EOT) from patients recruited to PALOMA-3 showed that *ESR1* mutations had a greater suppression by fulvestrant plus placebo compared to PIK3CA mutations but does not predict improvement in PFS on fulvestrant [56]. However, *ESR1* mutations that developed under selective aromatase inhibitor pressure were frequently subclonal, therefore limiting the potential for their dynamics to predict clinical outcome. Of note, in this study, changes in *ESR1* mutation abundance on treatment relative to baseline could not predict PFS in patients treated with palbociclib. Comparing *ESR1* mutation between baseline and EOT, 25.8% of *ESR1* mutant patients showed undetectable levels of mutations at the EOT. Patients with *ESR1* mutation clearance at day 15 more frequently showed no detectable levels of mutations at the EOT. Intriguingly, clearance at the EOT occurred more frequently in patients receiving palbociclib compared to placebo [56]. Analysis of mutations in PIK3CA, another possible biomarker, was also undertaken. PIK3CA mutations have been reported to be an early event in ER+ breast cancer and are found in more than 30% of ER+ primary treatment-naïve breast cancers. The frequency of PIK3CA mutations does not change under the pressure of endocrine treatments or the development of endocrine resistance and metastatic disease [22,57]. A decrease in PIK3CA ctDNA level after 15 days of treatment predicted a better PFS on palbociclib and fulvestrant treatment [56].

Recently published data evaluating OS in an exploratory analysis of PALOMA-3 showed that in patients receiving combination therapy, those with *ESR1* mutations had a longer OS compared to those with *ESR1* wild-type, albeit without reaching statistical significance (11.0 versus 4.7 months; *p* = 0.60). In this study, OS was similar in patients with or without PIK3CA mutations [58]. Analysis of ctDNA samples derived from patients enrolled in MONALEESA-2 detected *ESR1* in only 4% of cases; too low an incidence to reliably correlate to clinical outcome on ribociclib (Table 1) [58]. MONALEESA-2 is a phase III trial, which tested ribociclib plus anastrozole versus anastrozole alone, in first-line treatment of postmenopausal patients with either de novo ER+/HER2-negative MBC or with previously early-stage disease that progressed at least 12 months subsequent to the last dose of adjuvant AI therapy. This inclusion criteria therefore selected patients with a high likelihood of endocrine-sensitive disease (given the absence of prior drug exposure in the de novo patients, and a durable response in those previously exposed). As such, it is not surprising that a low percentage of *ESR1* mutations was observed in this cohort.

Overall, these data suggest that baseline *ESR1* and PIK3CA mutations do not hold predictive value as biomarkers for CDK4/6 inhibitor therapy [50]; however, dynamic changes in mutational ctDNA on treatment could itself be a biomarker of response or resistance to CDK4/6 inhibitor treatment [56]. 

## 5. Potential New Therapeutic Agents and Strategies

New SERMs or SERDs (with or without CDK4/6 inhibition) or high-dose tamoxifen or fulvestrant may represent possible therapeutic strategies to overcome resistance linked to *ESR1* mutations. A short summary of the properties of new compounds under development is presented in Table 2, and has also been discussed in greater detail elsewhere [59]. Completed and ongoing clinical trials involving potential new agents and strategies are presented in Table 3. Among these new compounds are bazedoxifene [60] and brilanestrant [61], with pre-clinical studies demonstrating efficacy in inhibiting cellular growth in models harboring *ESR1* mutations. Bazedoxifene—a third generation SERM with SERD activity—effectively arrested BC cell growth, regardless of whether cells were sensitive or resistant to prior tamoxifen treatment. Bazedoxifene administration triggers a proteasomal degradation of ER by altering its conformation [60]. Similarly, brilanestrant, a novel selective SERD, has demonstrated strong inhibitory activity in tamoxifen-sensitive and -resistant metastatic BC cells [62,63]. Recently, Bahreini et al. conducted pre-clinical testing of the new orally-active SERD AZ9496 and brilanestrant. Both were able to inhibit growth in *ESR1* wild-type and *ESR1*-mutated BC cells, including Y537S mutants [30]. Additionally, AZ9496 also reduced cell growth in xenograft models with *ESR1* D538G mutations [64]. This study highlighted the need to include assessment of specific mutations, given that each mutation is different—in particular, Y537S mutations may require higher doses of drugs to reach full inhibition [64].

Lasofoxifene is another new SERM associated with positive pre-clinical data. Laine and colleagues have assessed the efficacy of lasofoxifene in pre-clinical models [66]. Mice were injected with three different MCF7 variants (MCF7 wild type Y537S, and D538G) and treated with vehicle, fulvestrant, or three different doses of lasofoxifene (1, 5, 10 mg/kg; 5 days/week). Compared to fulvestrant, lasofoxifene was more effective at 5 and 10 mg/kg in the Y537S tumor. Further studies have also tested novel combination strategies incorporating new SERDs or SERD hybrids (SSH) as single agents, or in combination with CDK4/6 inhibitors in *ESR1*-mutated MCF7 cells and animal models, demonstrating that such a combination approach may prolong the length of treatment response, compared to single-agent therapy [37].

A recently completed phase I clinical trial tested AZD9496 in advanced ER+/HER2-negative BC (NCT02248090). The primary endpoint was to find the maximum tolerated dose, and to assess safety and drug activity in patients with and without *ESR1* mutations. Early modifications in circulating tumor cells (CTCs) and ctDNA of patients recruited to that trial have recently been reported, assessing the significance that these changes may have in relation to the pharmacodynamics and efficacy of AZD9496 [68]. Patients who had ≥5 CTCs per 7.5 mL of whole blood at baseline had worse PFS than those with <5 CTCs per 7.5 mL (*p* = 0.0003). *ESR1* mutational status at baseline was not associated with prognosis, whereas persistence of high *ESR1* mutational ctDNA at day 15 of treatment with AZD9496 was related to worse PFS (*p* = 0.0007).

Another phase Ib/II single arm clinical trial has been conducted in patients with ER+/HER2-negative MBC, assessing the efficacy of palbociclib given in combination with basedoxifene (NCT02448771). Participants were required to have had progressive disease after one or more lines of endocrine therapy, and up to two chemotherapy lines in the metastatic setting to be considered eligible for study entry. All patients (*N* = 36) were CDK4/6 inhibitor-naïve. Preliminary analyses showed a clinical benefit rate of 39% at 24 weeks, with the combination proving to be well-tolerated. Analysis of ctDNA is not yet available (Table 2).

Pre-clinically, SAR439859, a new oral non-steroidal SERD, has shown potent ER-degrading and -antagonist activity that results in strong inhibition of ER signaling in various ER+ BC cell lines, including cell models harboring *ESR1* mutations [70]. An ongoing trial is testing the activity and safety of SAR439859 in postmenopausal patients with ER+ MBC, both as a single agent and in combination with palbociclib (NCT03284957). A further phase II study is ongoing to evaluate the efficacy of SAR439859 compared to physician’s choice therapy, in pre- and postmenopausal women with ER+ MBC (NCT04059484).

Elacestrant is a SERD which has been shown to induce degradation of ER, inhibit ER-mediated signaling and growth of ER+ BC cell lines in vitro and in vivo, and inhibit tumor growth in multiple patient-derived xenograft (PDX) models, including demonstrable anti-tumor activity both when applied as a single agent and in combination with palbociclib in two PDX BC models harboring *ESR1* mutations [73]. Currently, elacestrant is under clinical investigation in postmenopausal women with ER+ MBC in two phase 1 studies. The first is a dose escalation/expansion study (NCT02338349), and the second will assess the efficacy of elacestrant and its effect on pharmacodynamic endpoints, including an evaluation of its influence over the availability of ER binding sites in MBC lesions assessed with 16α-18F-Fluoro-17β-Estradiol positron emission tomography imaging (NCT02650817). The last is EMERALD (NCT03778931), an international multicentre phase III study, which will compare the efficacy and safety of elacestrant to physician’s choice endocrine monotherapy (an AI or fulvestrant) in patients with *ESR1*-mutated ER+/HER2-negative metastatic BC. 

The experimental agent H3B-5942 belongs to a class of orally-available ER covalent antagonists (SERCA). In vivo, H3B-5942 has shown antitumor activity as a single agent in BC xenograft models with *ESR1* wild-type and harboring *ESR1* Y537S mutations that was superior to fulvestant treatment [71]. The efficacy of H3B-5942 increased when given in combination with CDK4/6 inhibitors and mTOR inhibitors in *ESR1* wild-type and *ESR1* mutant BC models [71]. PADA -1 (“Palbociclib and Circulating Tumor DNA for ESR1 Mutation Detection”) (NCT03079011) is an ongoing trial designed to evaluate the efficacy of a switch in ET (AI changed to fulvestrant) combined with palbociclib at the time that *ESR1* mutations are detected in ctDNA on treatment [72]. This randomized, open-label, multicenter, phase III trial is enrolling patients to receive an AI plus palbociclib as first-line therapy for ER+/HER2-negative MBC. Patients are screened for *ESR1* mutations in ctDNA at regular intervals whilst on treatment. Patients who have increased ctDNA *ESR1* mutations in the absence of demonstrable progressive disease are randomized (1:1) to one of two arms: No change to existing therapy until disease progression as per RECIST criteria (Arm A), or a switch in endocrine therapy from an AI to fulvestrant, with palbociclib continued (Arm B). Furthermore, cross-over of 80 patients from Arm A following progression is mandated by the study protocol. This study will clarify if *ESR1* holds a predictive and a prognostic value in patients treated with CDK4/6 inhibitors.

Given the pre-clinical observation that *ESR1*-mutated disease may retain some sensitivity to tamoxifen or fulvestrant (albeit at a higher dose), re-challenging with these agents using different dosages or schedules may be attempted therapeutically. The CONFIRM study demonstrated that prolonged survival was associated with higher fulvestrant doses [74]; as such, it may be privy to investigate if high doses of fulvestrant can inhibit the growth of tumors that harbor *ESR1* mutations [75]. A small phase II clinical study has tested the safety and tolerability of high-dose tamoxifen in patients with prostate cancer (160 mg/m^2^/day) [76]. This regimen was well-tolerated, aside from Grade 3 neurotoxicity that occurred in 29% of patients, which was found to be rapidly reversible and adequately managed with dose modification. Furthermore, the efficacy and safety of tamoxifen 100 mg/day in metastatic breast cancer is currently being evaluated in a recently completed phase II trial (NCT03045653). These may establish a pathway to clinically test higher doses of tamoxifen in patients with *ESR1*-mutated BC.

## 6. Conclusions

A major challenge remains in overcoming endocrine resistance in metastatic ER+ disease. *ESR1* mutations are recognized as a mechanism of endocrine therapy failure. *ESR1* mutations are infrequently seen in primary BC, occurring at a rate below 5%. However, in advanced BC, *ESR1* mutations occur at a frequency between 20 and 40%, dependent on assay techniques, and relative to the number of treatments lines received in the advanced setting. 

ctDNA has potential for the non-invasive monitoring of tumor mutational status over time and treatments [38], and as such, collecting and analyzing plasma samples in patients with metastatic disease could prove useful in observing disease behavior [41]. Early changes in circulating tumor DNA (ctDNA) levels may reflect early response to treatment, but the impact of tumor heterogeneity is still unknown [56]. Several studies have identified hotspot mutations in LBD *ESR1* (Y537S, D538G, and E380Q), which confer constitutive ligand-independent activity. Pre-clinical studies suggest that BC cells harboring LBD *ESR1* mutations confer partial resistance to tamoxifen and fulvestrant in vitro [2,28]. Moreover, other data suggest that LBD *ESR1* mutations confer complete resistance to aromatase inhibitor treatment [22].

*ESR1* mutations may be seen as a novel biological predictor of endocrine resistance. Indeed, in several research studies conducted in BC patients, the presence of *ESR1* mutations predicted poor response to AI treatment, yet contrastingly, only Y537S mutations conferred relative resistance to fulvestrant treatment. Available data suggest that baseline *ESR1* mutations do not hold predictive value as biomarkers for CDK4/6 inhibitor therapy [53]; however, the dynamic changes in mutational ctDNA on treatment may be a biomarker of response or resistance to CDK4/6 inhibitor treatment. Ongoing trials are required to clarify the role of *ESR1* mutational status in patients treated with CDK4/6 inhibitors plus ET. Overall, *ESR1* mutations reflect an unfavorable prognostic value, regardless of treatment. New SERDs and SERMs are under investigation to overcome endocrine resistance. Pre-clinical studies of these drugs demonstrated efficacy in inhibiting cancer cell growth in models harboring *ESR1* mutations. These novel treatments can overcome resistance related to *ESR1* mutations, potentially offering a new therapeutic option for patients with ER+/HER2 negative MBC in the future.

## Figures and Tables

**Table 1:** *ESR1* mutation rate reported in selected metastatic breast cancer trials.

Sample Studies	Patients (*n* Substudy/Total *n* on Trial)	Comparator Trial Arms	Prevalence of *ESR1* Mutations
BOLERO-2 (NCT00863655)	541/724	Exemestane + everolimus vs. exemestane	28.8%
SOFeA (NCT00253422)	161/723	Fulvestrant + anastrozole vs. fulvestrant	39%
PALOMA-3 (NCT01942135)	195/521	Palbociclib + fulvestrant vs. fulvestrant	25.3%
MONALEESA-2 (NCT01958021)	494/668	Ribociclib + letrozole vs. letrozole	4%
FERGI (NCT01437566)	153/168	Pictilisib + fulvestrant vs. fulvestrant	37%

**Table 2:** New oral anti-estrogen compounds.

Agent	Agent Class	Mechanism of Action	Pharmacokinetics
Basedoxifene [60,65]	SERM/SERD hybrid	Binds to ERα with high affinity; regulates ERα turnover (“SERD-like” profile)	Major metabolic pathway: Hepatic glucuronidation, Little-to-no cytochrome P450-mediated metabolism Half-life: 30 h Major route of elimination: Gastrointestinal
Brilanestrant [62]	SERD	Degrades ERα and interrupts ERα signaling	No available published data
Lasofoxifene [66,67]	SERM	Binds to ERα, induces conformational changes of ERα, preventing coactivator recruitment	major metabolic pathway: P450-mediated metabolism (CYP2C9) Half-life: 116–150 h Major route of elimination: Gastrointestinal
AZD9496 [68,69]	SERD	Degrades ERα; binds and down-regulates mutant ERα, including D538G and Y537S mutations	Major metabolic pathway: P450-mediated metabolism (CYP2C8) Half-life: Rapid and biphasic decline following peak (0.99–1.99 h) Major route of elimination: Gastrointestinal
SAR439859 [70]	SERD	Binds ERα, inducing a conformational change that results in ERα degradation	No available published data
Elacestrant [71]	SERD	Dose-dependent ER degrader, inhibits estradiol-dependent induction of ER target gene transcription and cell proliferation in BC cells with wild-type and Y537S, D538G mutant ERα.	No available published data
H3b-5942 [72]	SERCA	Inactivates both wild-type and Y537S-mutated ERα by targeting Cys530, inducing a unique antagonist conformation	No available published data

Abbreviations: SERM, selective estrogen receptor modulator; SERD, selective estrogen receptor down-regulator; SERCA, selective estrogen receptor covalent antagonists; SERM/SERD hybrid, ERα, estrogen receptor alpha; BC, breast cancer.

**Table 3:** Completed and ongoing trials in ER+/HER2-negative metastatic breast cancer with a focus on ESR1 mutational status and new selective estrogen receptor down-regulator (ClinicalTrials.gov accessed on 21 October 2019).

Agent	Study Design	Estimated Enrollment	Primary Endpoint(s)	Status
AZD9496 (NCT02248090)	Phase I, open-label	45	Activity, tolerability, and safety of treatment	Completed
Tamoxifen (NCT030045653)	Phase I, open-label	32	CBR at 16 weeks	Completed
Basedoxifene (NCT02448771)	Phase Ib/II, open-label	36	CBR at 24 weeks	Active, not recruiting
Palbociclib plus AI/fulvestrant (NCT03079011)	Phase III, open-label	800	Safety until randomization/efficacy from randomization	Active, not recruiting
Elacestrant (NCT02338349)	Phase I, open-label	57	Dose-limiting toxicity	Active not recruiting
Elacestrant (NCT02650817)	Phase Ib, open-label	16	Effect of ER binding after elacestrant treatment	Active not recruiting
Elacestrant versus endocrine therapy (NCT03778931)	Phase III, open-label	466	PFS in patients with *ESR1* mutations	Recruiting
SAR439859 as monotherapy or plus palbociclib (NCT03284957)	Phase I/II, open-label, non randomized	224	Safety and efficacy of SAR439859 as monotherapy and in combination with palbociciclib	Recruiting
SAR439859 (NCT04059484)	Phase II, open-label randomized	282	PFS	Recruiting

Abbreviations: CBR, clinical benefit rate; PFS, progression free survival.
